# Supplementary figures and images for: Development of a gene doping detection method to detect overexpressed human follistatin using an adenovirus vector in mice
Source: PeerJ. 2021 Oct 20;9:e12285. doi: 10.7717/peerj.12285 (PMC8541302; doi:10.7717/peerj.12285)

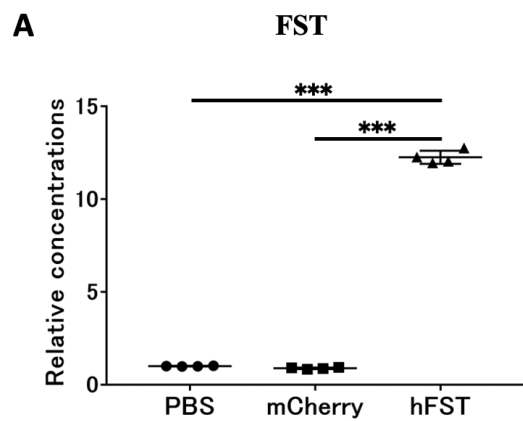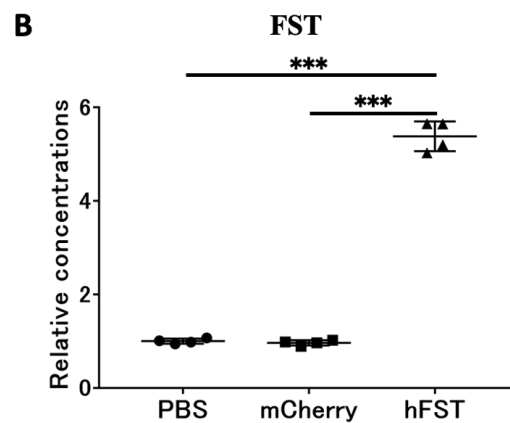

Supplement: Supplemental Information 1 — A; FST protein expression. B; FST protein expression in culture supernatant. To confirm the rAdV was completely working, cell experiments were conducted. FST protein was overexpressed in cell and secreted out of the cells in hFST group. Data are means ± SD.*p < 0.001. [file peerj-09-12285-s001.pdf]

**A**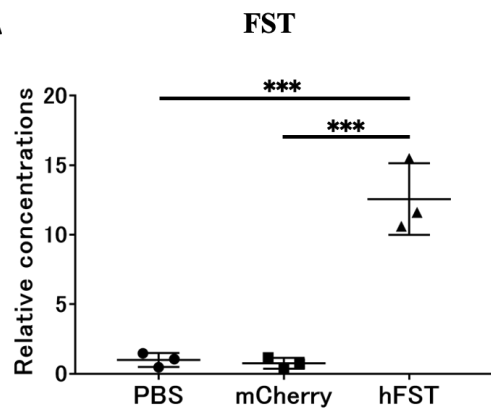**B**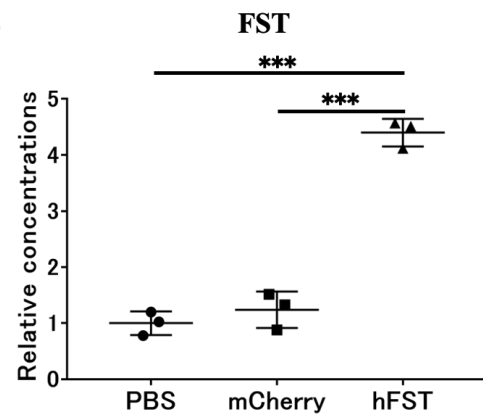

Supplement: Supplemental Information 2 — A; FST protein expression. B; FST protein expression in culture supernatant. To confirm the rAdV was completely working, cell experiments were conducted. FST protein was overexpressed in cell and secreted out of the cells in hFST group. Data are means ± SD.*p < 0.001. [file peerj-09-12285-s002.pdf]

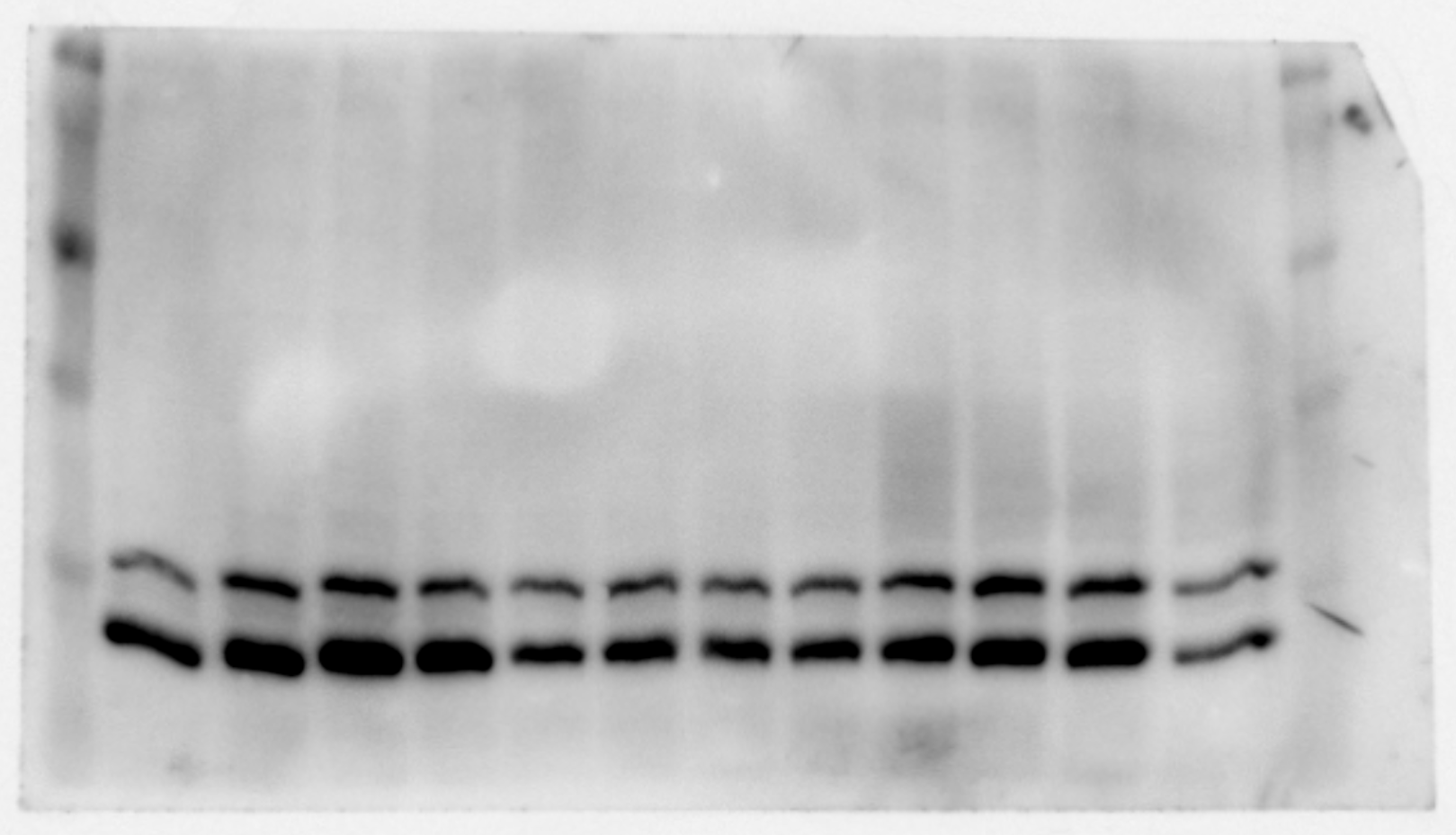

Supplement: Supplemental Information 5 [file peerj-09-12285-s005.zip › Supplemental Files_uncropped blots/Figure1/GAPDH_HuH7.png]

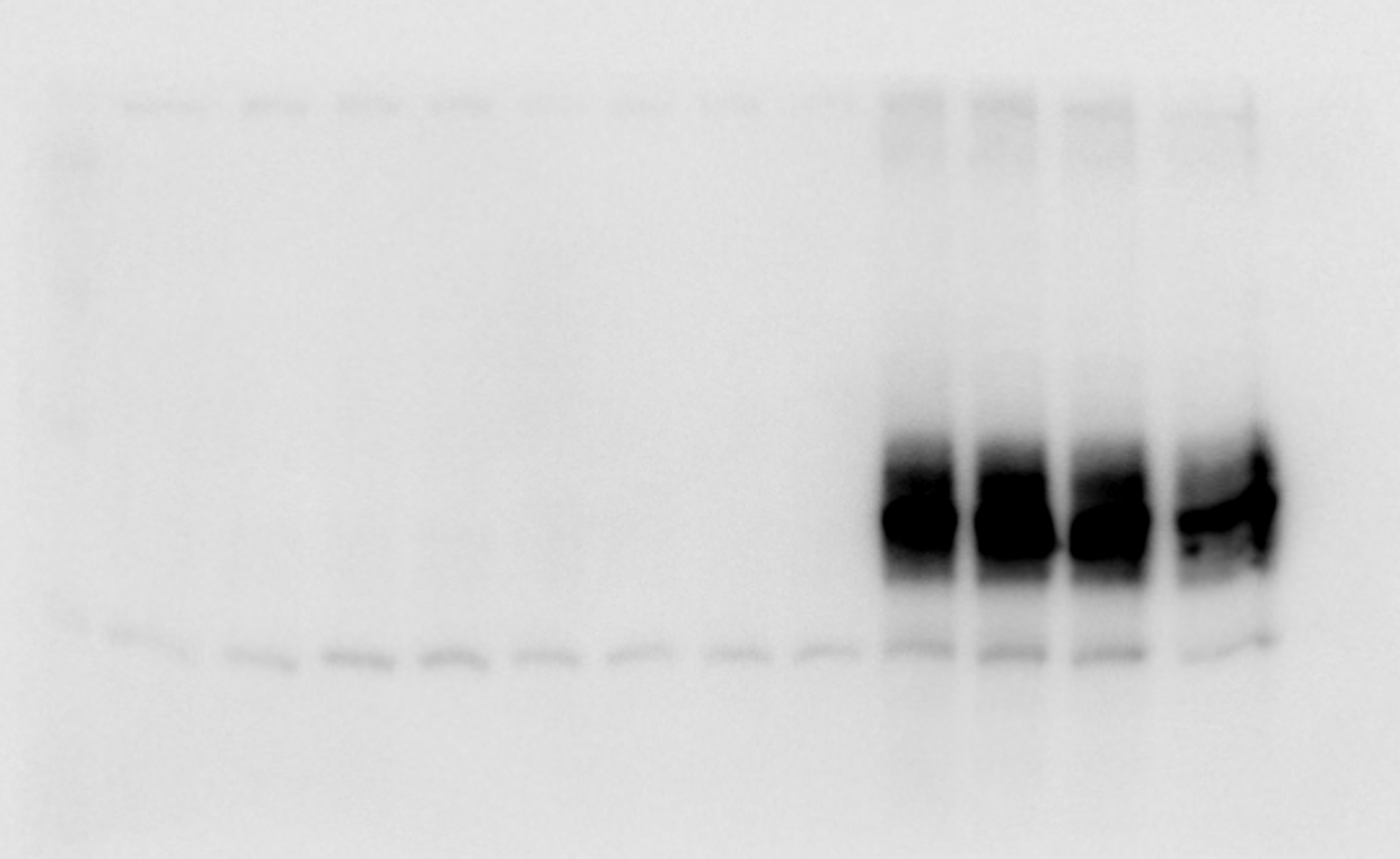

Supplement: Supplemental Information 5 [file peerj-09-12285-s005.zip › Supplemental Files_uncropped blots/Figure1/FST_HuH7.png]

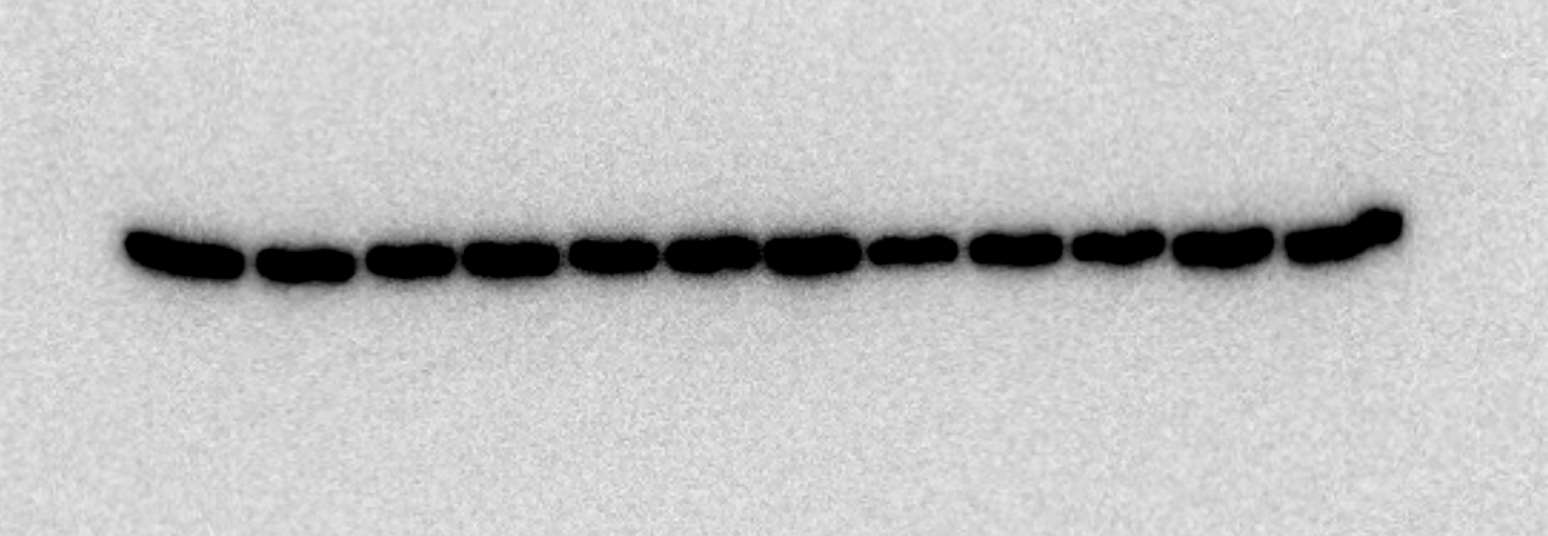

Supplement: Supplemental Information 5 [file peerj-09-12285-s005.zip › Supplemental Files_uncropped blots/Figure2/FST(Figure2-C)/B-Tubulin_C2C12.png]

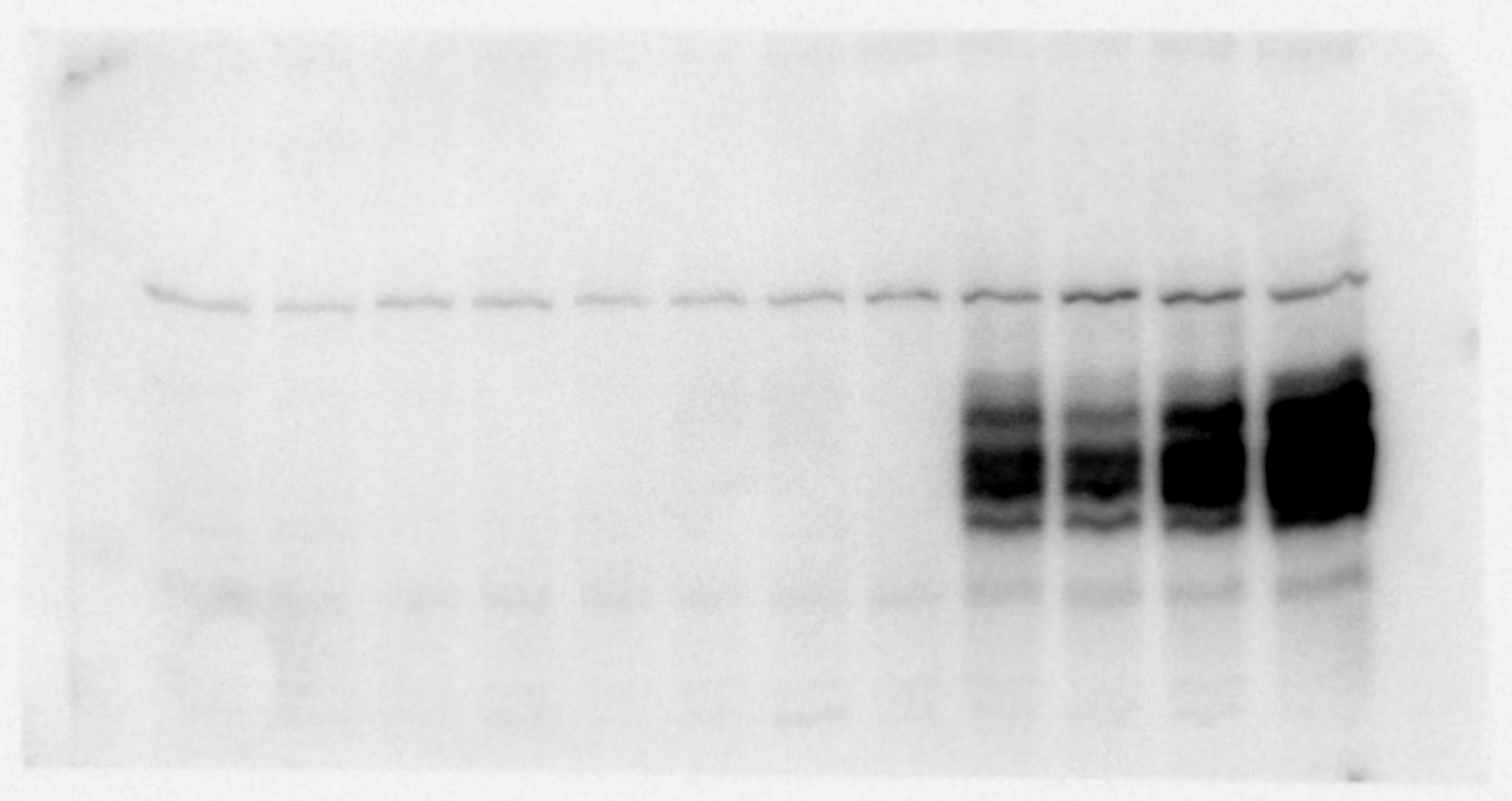

Supplement: Supplemental Information 5 [file peerj-09-12285-s005.zip › Supplemental Files_uncropped blots/Figure2/FST(Figure2-C)/FST_C2C12.png]

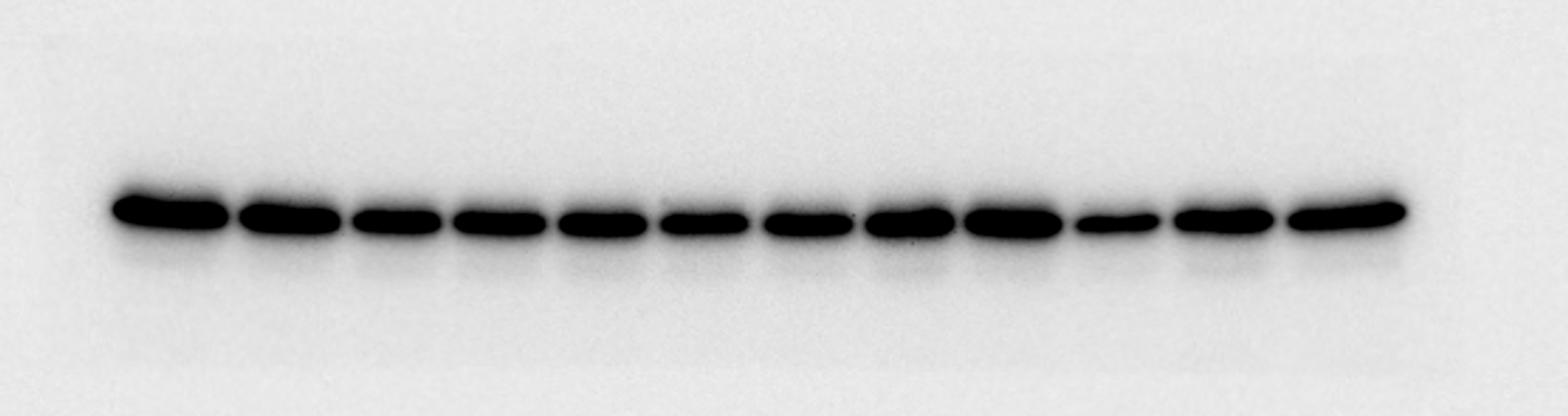

Supplement: Supplemental Information 5 [file peerj-09-12285-s005.zip › Supplemental Files_uncropped blots/Figure2/Akt(Figure2-E)/Akt_C2C12.png]

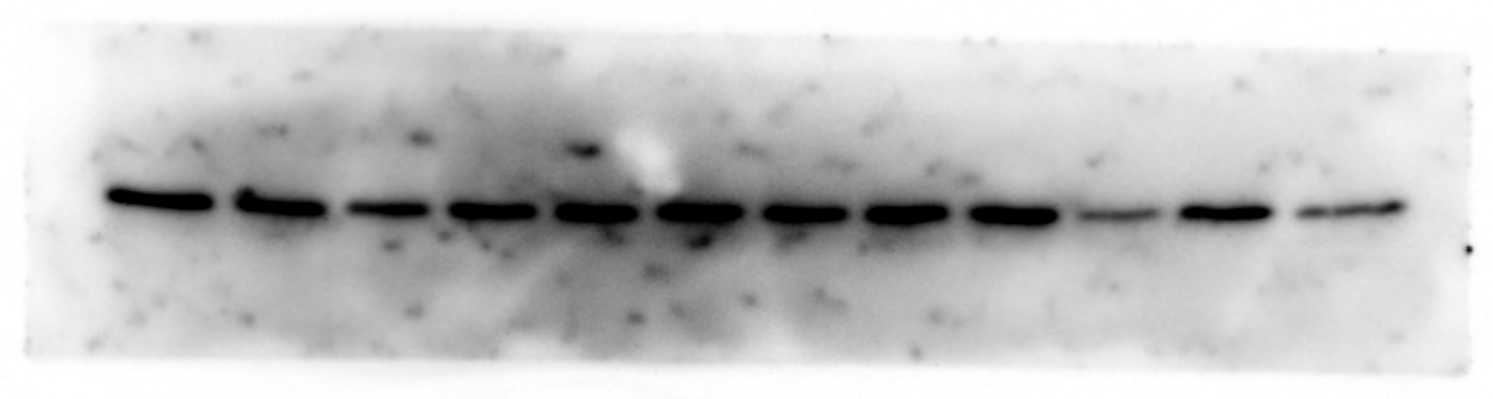

Supplement: Supplemental Information 5 [file peerj-09-12285-s005.zip › Supplemental Files_uncropped blots/Figure2/Akt(Figure2-E)/p-Akt_C2C12.png]

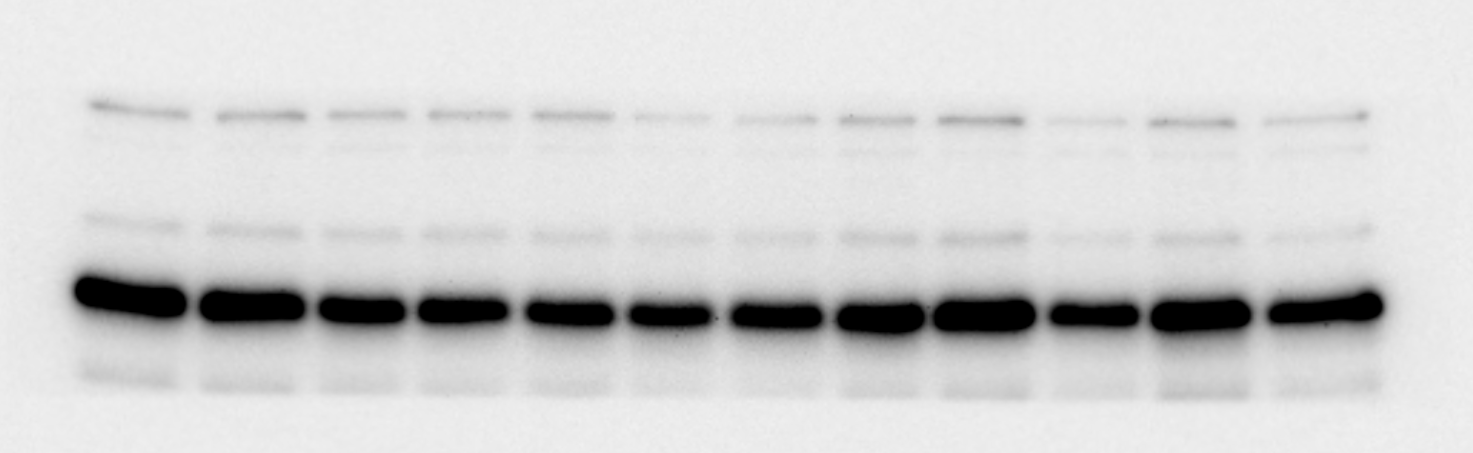

Supplement: Supplemental Information 5 [file peerj-09-12285-s005.zip › Supplemental Files_uncropped blots/Figure2/p70S6K(Figure2-F)/p70S6K_C2C12.png]

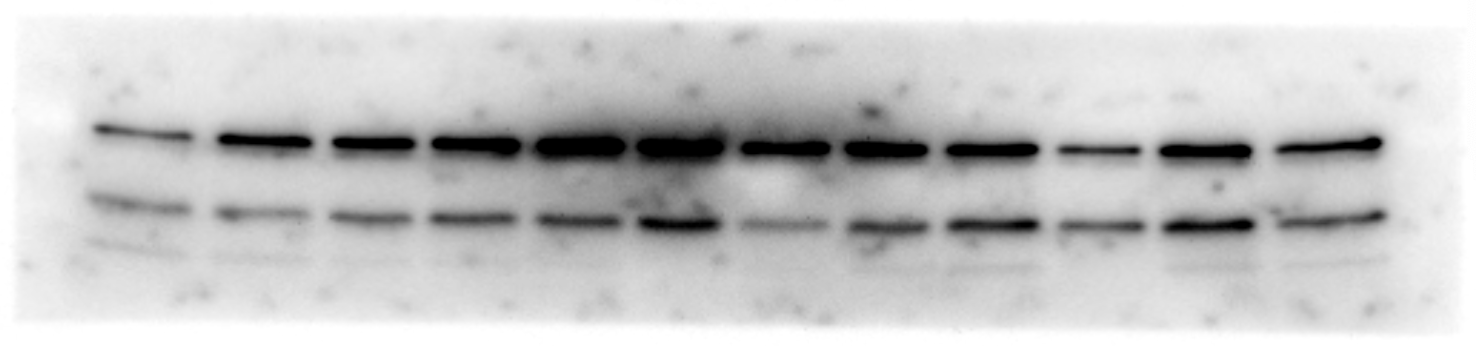

Supplement: Supplemental Information 5 [file peerj-09-12285-s005.zip › Supplemental Files_uncropped blots/Figure2/p70S6K(Figure2-F)/p-p70S6K_C2C12.png]
