# Supplementary material for: Development of a gene doping detection method to detect overexpressed human follistatin using an adenovirus vector in mice
Source: PeerJ. 2021 Oct 20;9:e12285. doi: 10.7717/peerj.12285 (PMC8541302; doi:10.7717/peerj.12285)
Supplement: Supplemental Information 3 [file peerj-09-12285-s003.docx]

| Manufacturer | Santa Cruz Biotechnology | Cell Signaling Technology | Proteintech Group | Cell Signaling Technology | Cell Signaling Technology | Cell Signaling Technology | Cell Signaling Technology | Cell Signaling Technology | Cell Signaling Technology |
| --- | --- | --- | --- | --- | --- | --- | --- | --- | --- |
| Catalog number | Sc-365003 | #2146 | 60004-1-lg | #9271S | #9272 | #9205S | #2708S | #7076 | #7074 |
| Species | Mouse | Rabbit | Mouse | Rabbit | Rabbit | Rabbit | Rabbit | Mouse | Rabbit |
| Antibody dilution | 1 / 100 | 1 / 2000 | 1 / 2000 | 1 / 1000 | 1 / 2000 | 1 / 1000 | 1 / 1000 | 1 / 1000 – 1 / 3000 | 1 / 2000 – 1 / 3000 |
| Antibody name | Follistatin | B-Tubulin | Glyceraldehyde-3-phosphate dehydrogenase (GAPDH) | p-Akt^Ser473^ | Akt | p-p70S6K^Thr389^ | P70S6K | Anti-mouse IgG, HRP-linked Antibody | Anti-rabbit IgG, HRP-linked Antibody |
