# Supplementary material for: Development of a gene doping detection method to detect overexpressed human follistatin using an adenovirus vector in mice
Source: PeerJ. 2021 Oct 20;9:e12285. doi: 10.7717/peerj.12285 (PMC8541302; doi:10.7717/peerj.12285)
Supplement: Supplemental Information 4 [file peerj-09-12285-s004.docx]

| Predicted Size (bp) | 147 | | | 188 | | | 146 | | 167 | | 165 | |
| --- | --- | --- | --- | --- | --- | --- | --- | --- | --- | --- | --- | --- |
| Sequence | GCCTGCTTCCTCTGAGCAATA | /56-FAM/TGGGCAGAT/ZEN/CTATTGGATTAGCC/3IABkFQ/ | TCTTCACAGGACTTTGCTTTGAT | TCATATGCCAAGTACGCCCC | /56-FAM/TGGGACTTT/ZEN/CCTACTTGGCAGTAC/3IABkFQ/ | CCCGTGAGTTCAAACCGCTAT | GCGATGAGCTGTGTCCTGA | GTTTTACAGGCAGATTCAGTTGC | CACTGGTCTAGGACCCGAGAA | AGGGGGAGATGTTCAGCATGT | CTCAACACGGGAAACCTCAC | CGCTCCACCAACTAAGAACG |
|  | Forward | Probe | Reverse | Forward | Probe | Reverse | Forward | Reverse | Forward | Reverse | Forward | Reverse |
| Target | Human Follistatin (hFST) | | | Cytomegalovirus promoter  (CMVp) | | | Mouse Follistatin (mFST) | | Acid ribosomal phosphoprotein  (36B4) | | GAPDH | |
